# *Calanthe* x*hsinchuensis* (Orchidaceae), a new natural hybrid from Taiwan

**DOI:** 10.1186/1999-3110-54-25

**Published:** 2013-08-30

**Authors:** Yung-I Lee

**Affiliations:** Biology Department, National Museum of Natural Science, No 1, Kuan-Chien Rd.,, Taichung, Taiwan

**Keywords:** *Calanthe arisanensis*, *Calanthe sieboldii*, *Calanthe* x*hsinchuensis*, *Calanthe*, Taiwan, Chromosome number, Natural hybrid, Orchidaceae

## Abstract

**Background:**

Natural hybridization in plants is a vital mechanism of speciation. *Calanthe arisanensis* and *C. sieboldii* occur in the same habitat in northern Taiwan, where there are a number of plants whose morphologically characters are similar in between of these two species. In this report, a new natural hybrid, *Calanthe* species – *C.* x*hsinchuensis* Y.I. Lee putatively derived from the natural hybridization of *C. arisanensis* and *C. sieboldii* was described and illustrated. Besides, somatic chromosome number was counted.

**Results:**

The morphological and histological data of flowers, capsules, roots and leaves of *C.* x*hsinchuensis* show an intermediate condition between its putative parents. The chromosome number, 2n = 40, is also congruent with its putative parents. Color plates and line drawings are provided to aid in identification.

**Conclusion:**

Base on the morphological characteristics of reproductive and vegetative organs, and the distributions of putative parents, it is proposed that *C.* x*hsinchuensis* is a natural hybrid between *C*. *arisanensis* Hayata and *C*. *sieboldii* Decaisne *ex* Regel.

**Electronic supplementary material:**

The online version of this article (doi:10.1186/1999-3110-54-25) contains supplementary material, which is available to authorized users.

## Background

Natural hybridization is an important mechanism of plant evolution (Arnold, 1997). Fertile hybrids may intercross or backcross with parental species, producing abundant genetic and phenotypic variation for natural selection. The genus *Calanthe* comprises more than 170 species widely distributed from Africa, Asia, Pacific Islands and Australia (Jin and Li, 2007). This genus is characterized by pubescent roots, often clustered leaves, petals and sepals similar, spurred lip and eight pollinia. In Taiwan, there are 19 species of *Calanthe*(Su, 2000; Flora of Taiwan). *Calanthe arisanensis* is an endemic species with pinkish white flowers that could be found throughout the island at the altitudes about 1000 meter (Lin, 1988;Su, 2000). *Calanthe sieboldii* is closely related to *Calanthe arisanensis* with bright yellow flowers that could be found in the northern areas of Taiwan (Lin, 1988;Su, 2000). *C. sieboldii* is also distributed in Ryukyu Islands, southern Japan and Korea. In northern Taiwan, *C. arisanensis* and *C. sieboldii* occur in the same habitat, and they both bloom during March and April. In some natural habitats, besides these two *Calanthe* species, there are a number of plants whose morphologically characters are similar in between of *C. arisanensis* and *C. sieboldii* that are considered to be their natural hybrid. In this report, a new *Calanthe* species putatively derived from the natural hybridization of *C. arisanensis* and *C. sieboldii* was documented.

## Methods

### Material

Specimens of *Calanthe* x*hsinchuensis*, *C. arisanensis* and *C. sieboldii* were collected from Jiashih Township of Hsinchu County, Taiwan and cultivated in the greenhouse at National Museum of Natural Science, Taiwan for further morphological comparison, SEM, histological and cytological studies. The vouchers are deposited at TNM.

### Chromosome preparation

The actively growing root tips were cut and pretreated in 2 mM 8-hydroxyquinoline at 25°C for 5 h to accumulate prometaphase cells, rinsed with distilled water, then fixed in fresh prepared Farmer’s fluid (three parts of ethanol to one part of glacial acetic acid). Root tips were macerated with 6% cellulose (Onoauka R-10, Yakukt Honsha, Japan) and 6% pectinase (Sigma Chemical Co., St. Louis, Mo.) in 75 mM KCl, pH = 4.0 at 37°C for 30 min. After a brief wash with 45% acetic acid solution, root tips were squashed as described by Aoyama (1989), then the chromosomes were stained with DAPI in an antifade solution (Vector Laboratories, CA, USA). The images were captured digitally by a CCD camera attached to a Zeiss Axioskop 2 microscope (Carl Zeiss AG, Germany).

### Scanning electron microscopy

The dry seeds were mounted on aluminum stubs with adhesive or silver paint, coated with 30 nm gold in a sputter coater and observed using a scanning electron microscope (S-3000 N; Hitachi, Japan).

### Histological observations

Root sections were cut and fixed in 2.5% glutaraldehyde and 1.6% paraformaldehyde buffered with 0.05 M phosphate buffer, pH 6.8, for 24 hours at 4°C. After fixation, the samples were rinsed in three 15-min changes of buffer. The materials were dehydrated in an acetone series and embedded in the Spurr’s resin (Electron Microscope Sciences, Washington, PA). Sections of 1 μm were obtained by a glass knife on an ultramicrotome (Leica, Wetzlar, Germany), and were stained with 0.1% alkaline TBO for 1 min at 60°C on a hot plate. The sections were viewed and the images were captured digitally using a CCD camera attached to a light microscope (Carl Zeiss AG).

## Results and discussion

### Taxonomic treatment

#### Calanthe xhsinchuensis

**Figure 1:**
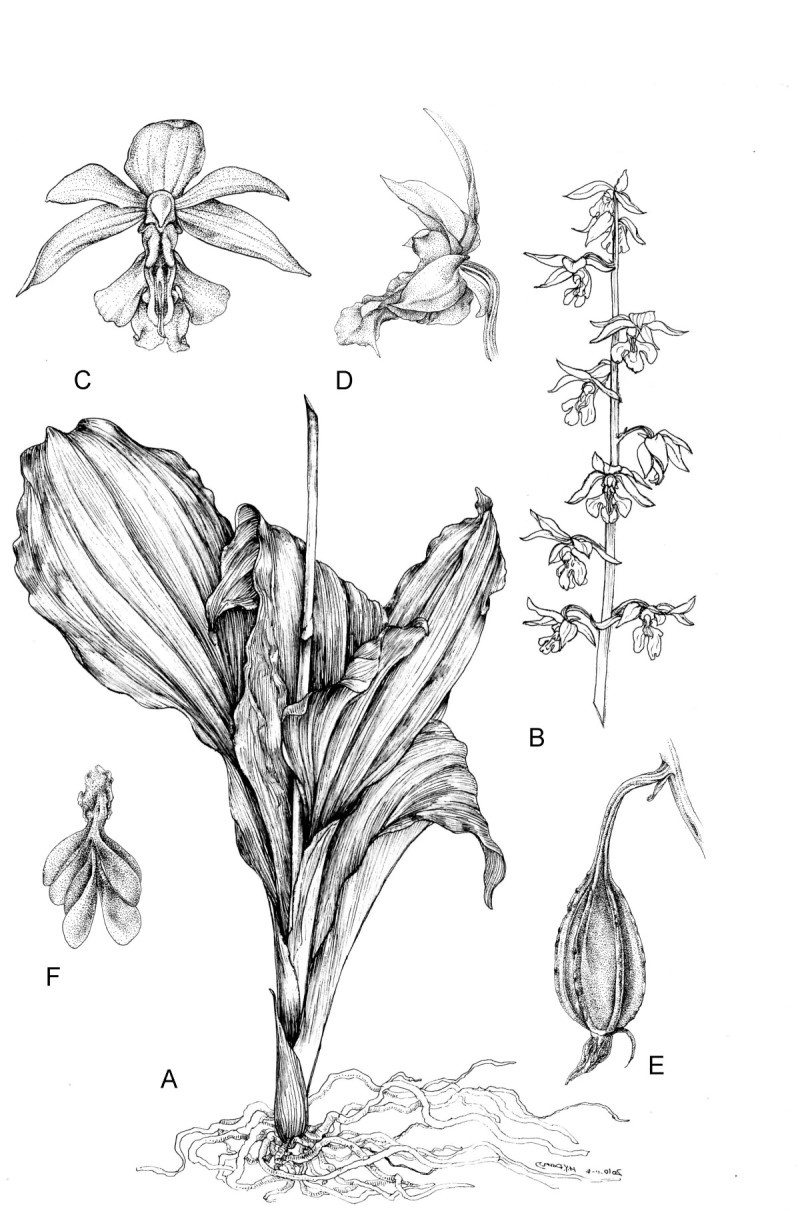
***Calanthe*** **x*hsinchuensis*** **.** **(A)** Plant; **(B)** Inflorescence; **(C)** Front view of flower; **(D)** Lateral view of flower; **(E)** Capsule; **(F)** Pollinia.

**Figure 2:**
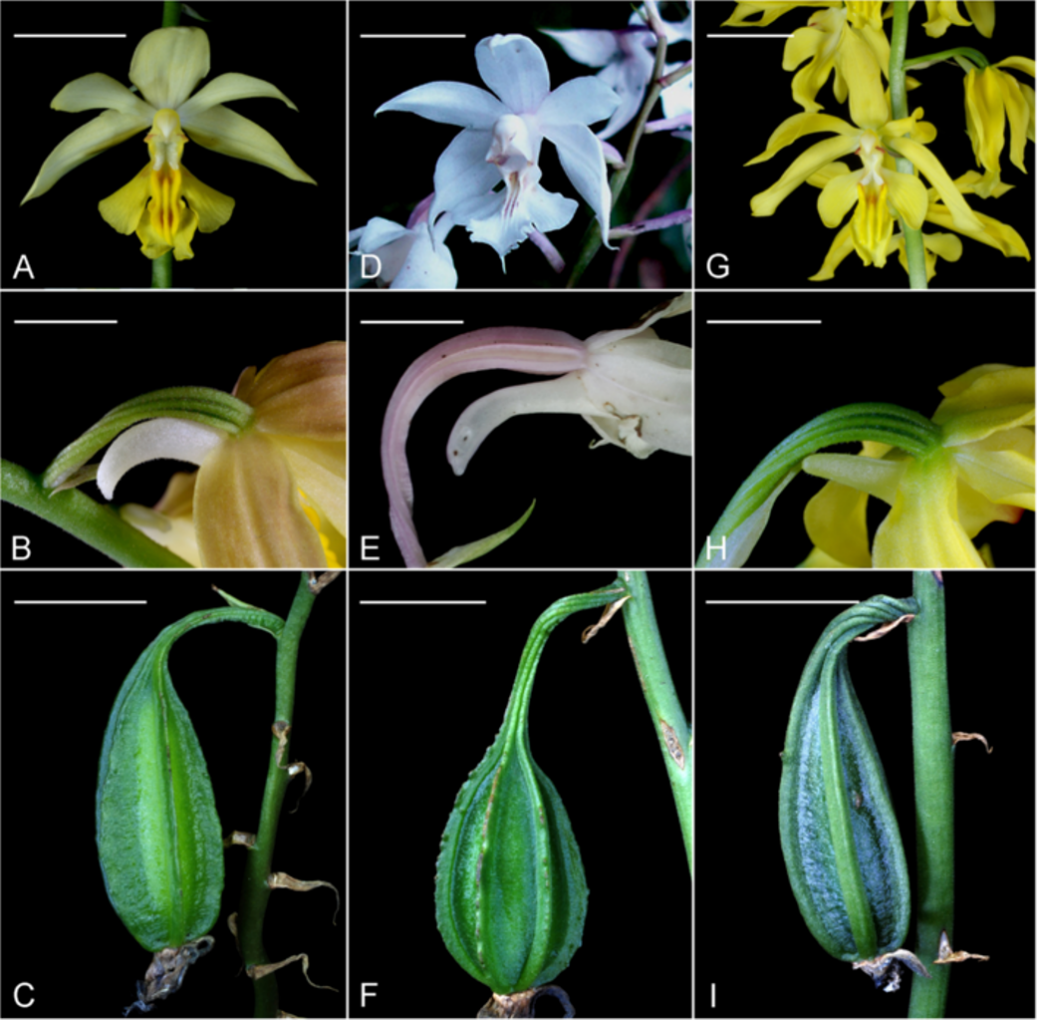
**Comparison of** ***Calanthe*** **x*hsinchuensis*** **(A-C) with putative parents,** ***C*** **.** ***arisanensis*** **(D-F) and** ***C*** **.** ***sieboldii*** **(G-I).** **(A**, **D** and **G)** Flowers, Scale bar = 2 cm; **(B**, **E** and **H)** Spur and ovary, Scale bar = 8 mm; **(C**, **F** and **I)** Capsule, Scale bar = 5 cm.

Y.I. Lee, *hybr. nov.*—TYPE: TAIWAN, Hsinchu County, Jiashih Township, Niao Zui Shan, elev. ca. 1,500 m, 25 Mar 2008, *Yung-I Lee 200902* (holotype: TNM). 新竹根節蘭. Figures 1 and 2.

#### Diagnosis

Hybrida natunali e C. arisanensi et C. sieboldii genita, forma foliorum, diam. radicum, labellis, calcaribus et fructibus omnino inter C. arisanensem et C. sieboldii, ab ambabus plantis 40–60 cm altis, floribus 4–5 cm diam, cremeo-flavidis differt.

#### Terrestrial

**Figure 3:**
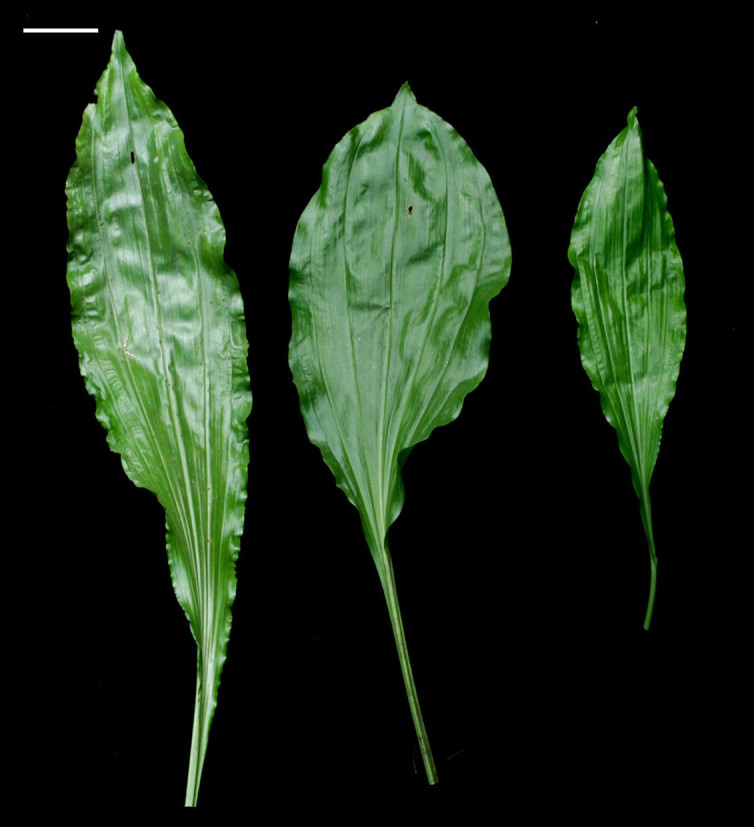
**Comparison of leaves of** ***Calanthe*** **x*hsinchuensis*** **(left) with putative parents,** ***C*** **.** ***sieboldii*** **(middle) and** ***C*** **.** ***arisanensis*** **(right).** Scale bar = 3 cm.

**Figure 4:**
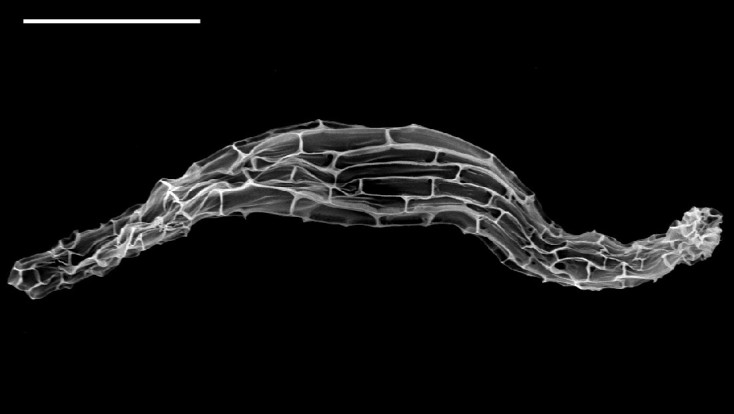
**The SEM microphotograph of** ***C.*** **x*hsinchuensis*** **seed.** Scale bar = 200 μm.

**Figure 5:**
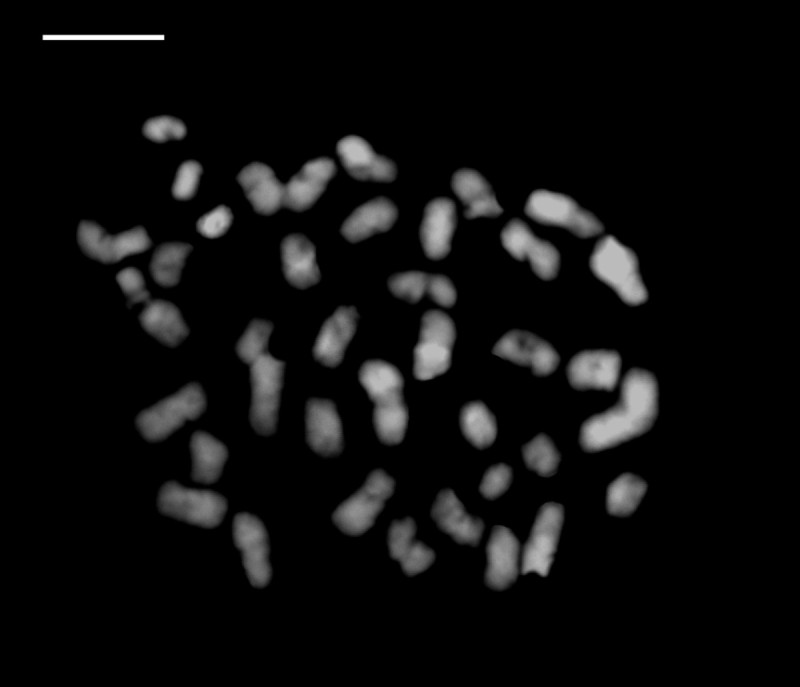
**Mitotic chromosome of** ***Calanthe*** **x*hsinchuensis*** **(2n = 40).** Scale bar = 10 μm.

Plants 40 – 60 cm tall. Pseudobulb globose-ovoid, 3 cm long, 2 cm in diam, with 3 or 4 nodes. Leaves 3 – 4, elliptic to narrow elliptic, apex acuminate, leaf blade 35 – 40 cm long, 6.5 - 9 cm wide, not fully opened at anthesis, petiole 5 – 10 cm long (Figure 3). Inflorescence raceme with 7 to 10 flowers arising with developing leaves and pseudobulbs, about 50 – 70 cm long, 4.2 – 4.9 cm wide; pedicel with ovary light green, about 2 cm long, ovary shortly pubescent; floral bracts lanceolate, 1 – 1.5 cm long. Flowers 4 – 5 cm across, creamy yellow; lip with brighter yellowish lobes. Dorsal sepal lanceolate-ovate, 2.5 – 3 cm long, 1.2 cm wide, acuminate at apex, slightly contracted at base; lateral sepals similar, slightly oblique, 2.7 – 3.2 cm long, 1.2 cm wide. Petals oblanceolate, 2 – 2.4 cm long, 0.5 cm wide, acuminate at apex. Lip deeply 3-lobed, 2 cm across, adnate to column at base, spurred; lateral lobes, broadly flabellate or ovate; central lobe oblong, margins undulate, apex emarginate with short aristate; disc with 2 short ridges and 3 slender, long ridges, the basal disc adjacent to column with 2 rows of dense white hairs. Spur white, curved, shortly pubescent 8 – 12 mm long. Column stout, 7 mm long. Anther 5 mm long, cordate; pollinia clavate, orange, 2 mm long, with short caudicles attached to viscidium; stigma solitary; rostellum bifid, acute at apex. Capsule with 6 lightly longitudinal ridges, 4.8 – 5.2 cm long, 1.6 – 2 cm wide. The seeds numerous, fusiform with white and transparent seed coat. The seed size about 750 μm long, 100 μm across (Figure 4). The somatic chromosome number of C. x*hsinchuensis* is 2n = 40 that is the same as its putative parents (Tanaka et al., 1981) (Figure 5).

**Table 1:** **Comparison of** ***Calanthe*** **x** ***hsinchuensis*** **with putative parents,** ***C*** **.** ***arisanensis*** **and** ***C*** **.** ***sieboldii***

	C. x***hsinchuensis***	***C***. ***arisanensis***	***C***. ***sieboldii***
Root diameter (mm)	1.9 – 2.1	2.1 – 2.5	1.5 – 1.6
Leaf			
Leaf shape	Elliptic to narrow elliptic	Narrowly elliptic	Broadly elliptic
Length (cm)	40 – 55	15 – 25	35 – 40
Width (cm)	6.5 - 9	4 – 4.5	9 – 12
Inflorescence			
Length (cm)	50 – 70	25 – 40	50 – 80
Width (mm)	4.2 – 4.9	3.1 – 3.2	4.9 – 5.1
Flower			
Tepal color	Creamy yellow	Pinkish white	Yellow
Spur (mm)	8 – 12	14 – 15	4 – 5
Spur shape	Curved	Curved	Antrorse
Capsule			
Capsule shape	Capsule with 6 lightly longitudinal wing-like ridges	Capsule with 6 longitudinal wing-like ridges	Capsule without conspicuous longitudinal wing-like ridges
Length (cm)	4.8 – 5.2	5.2 – 6.1	5.1 – 5.3
Width (cm)	1.6 – 2.0	2.0 – 2.3	1.5 – 1.7

#### Distribution

It is known mainly from the mountain area in Jiashih Township (1500 m) of Hsinchu County in northern Taiwan.

#### Etymology

The name of this species is derived from the Hsinchu County, the place of its discovery in northern Taiwan.

#### Notes

**Figure 6:**
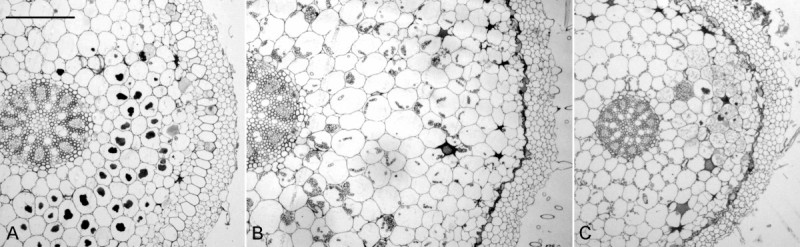
**The cross sections of** ***Calanthe*** **roots. (A)** *Calanthe* x*hsinchuensis*; **(B)** *C*. *arisanensis*; **(C)** *C*. *sieboldii*. Scale bar = 3 mm.

*C.* x*hsinchuensis* only occurs at the sites where both *C. arisanensis* and *C. sieboldii* could be found together. Base on the comparison of morphological characteristics of reproductive and vegetative organs of *C.* x*hsinchuensis* and its putative parental species (Table 1, Figures 2, 3 and 6), it is proposed that this unusual plants occur in Jiashih Township of Hsinchu County is a natural hybrid between *C*. *arisanensis* and *C*. *sieboldii*.

The root diameter of *C.* x*hsinchuensis* shows an intermediate condition between its putative parents (Table 1; Figure 6). The root diameter of *C*. *arisanensis* (Figure 6B) is the thickest among the three species, whereas the root diameter of *C*. *sieboldii* (Figure 6C) is the thinnest. The velamen of *C.* x*hsinchuensis* is 4 to 5 celled-wide, and the cortex is 8 to 9 celled-wide (Figure 6A). The hyphae, and pelotons could be found in the cells of the cortex. The other features, including leaf shape (Figure 3) and flower color (Figures 2A,D and G) of *C.* x*hsinchuensis* are intermediate between those of *C*. *arisanensis* and *C*. *sieboldii*. The spur shape of *C.* x*hsinchuensis* is curved that is similar to the spur shape of *C*. *arisanensis*; while the ovary color of *C.* x*hsinchuensis* is light green that is similar to the ovary color of *C*. *sieboldii* (Figures 2B,E and H). The capsule shape of *C*. *arisanensis* is characterized by having 6 longitudinal wing-like ridges, while *C*. *sieboldii* has no conspicuous wing-like ridges on its capsule. *C.* x*hsinchuensis* has 6 lightly longitudinal ridges on the capsule, showing an intermediate condition (Figures 2C,F and I).

## Conclusion

All the available data and the distributions of putative parents support the recognition of the new species *C.* x*hsinchuensis* is a natural hybrid between *C*. *arisanensis* Hayata and *C*. *sieboldii* Decaisne *ex* Regel.

## Authors’ original submitted files for images

Below are the links to the authors’ original submitted files for images. Authors’ original file for figure 1 Authors’ original file for figure 2 Authors’ original file for figure 3 Authors’ original file for figure 4 Authors’ original file for figure 5 Authors’ original file for figure 6

## References

[CR1] Aoyama M (1989). Karyomorphological studies in *Cymbidium* and its allied genera. Orchidaceae. Hiroshima Bot. Gard. Bull.

[CR2] Arnold ML (1997). Natural Hybridization and Evolution.

[CR3] Jin XH, Li H (2007). A new species of *Calanthe* (Orchidaceae) from Yunnan. China Nord J Bot.

[CR4] Lin TP (1988). Native Orchid Species of Taiwan vol. 2.

[CR5] Su HJ, Huang TC (2000). Orchidaceae. Flora of Taiwan.

[CR6] Tanaka R, Karasawa K, Ishida G (1981). Karyomorphological observation on *Calanthe*. Japan Bull Hiroshima Bot Gard.

