# Supplementary figures and images for: Calanthe xhsinchuensis (Orchidaceae), a new natural hybrid from Taiwan
Source: Bot Stud. 2013 Aug 30;54:25. doi: 10.1186/1999-3110-54-25 (PMC5430342; doi:10.1186/1999-3110-54-25)

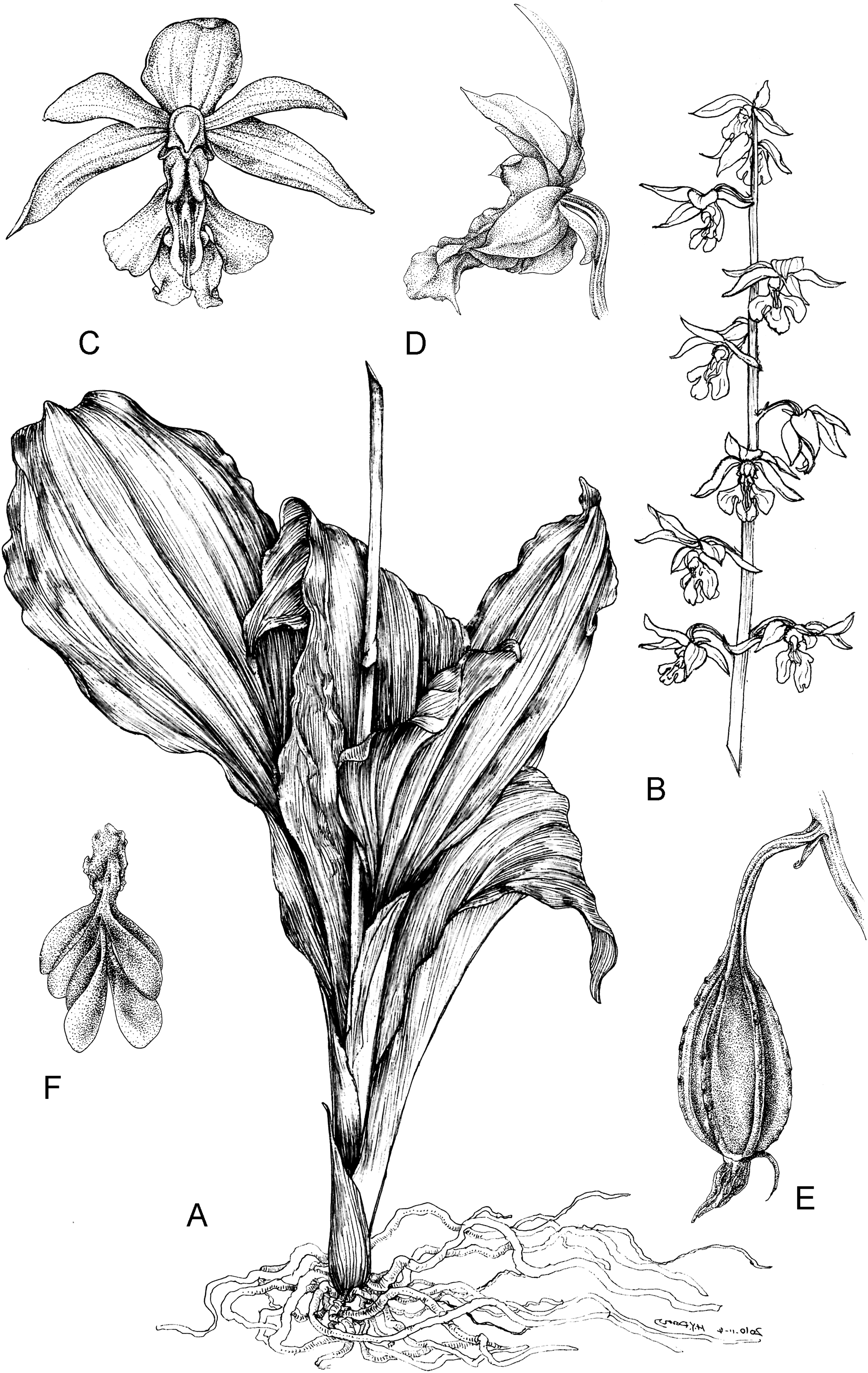

Supplement: Supplementary file 1 — Authors’ original file for figure 1 [file 40529_2011_22_MOESM1_ESM.tif]

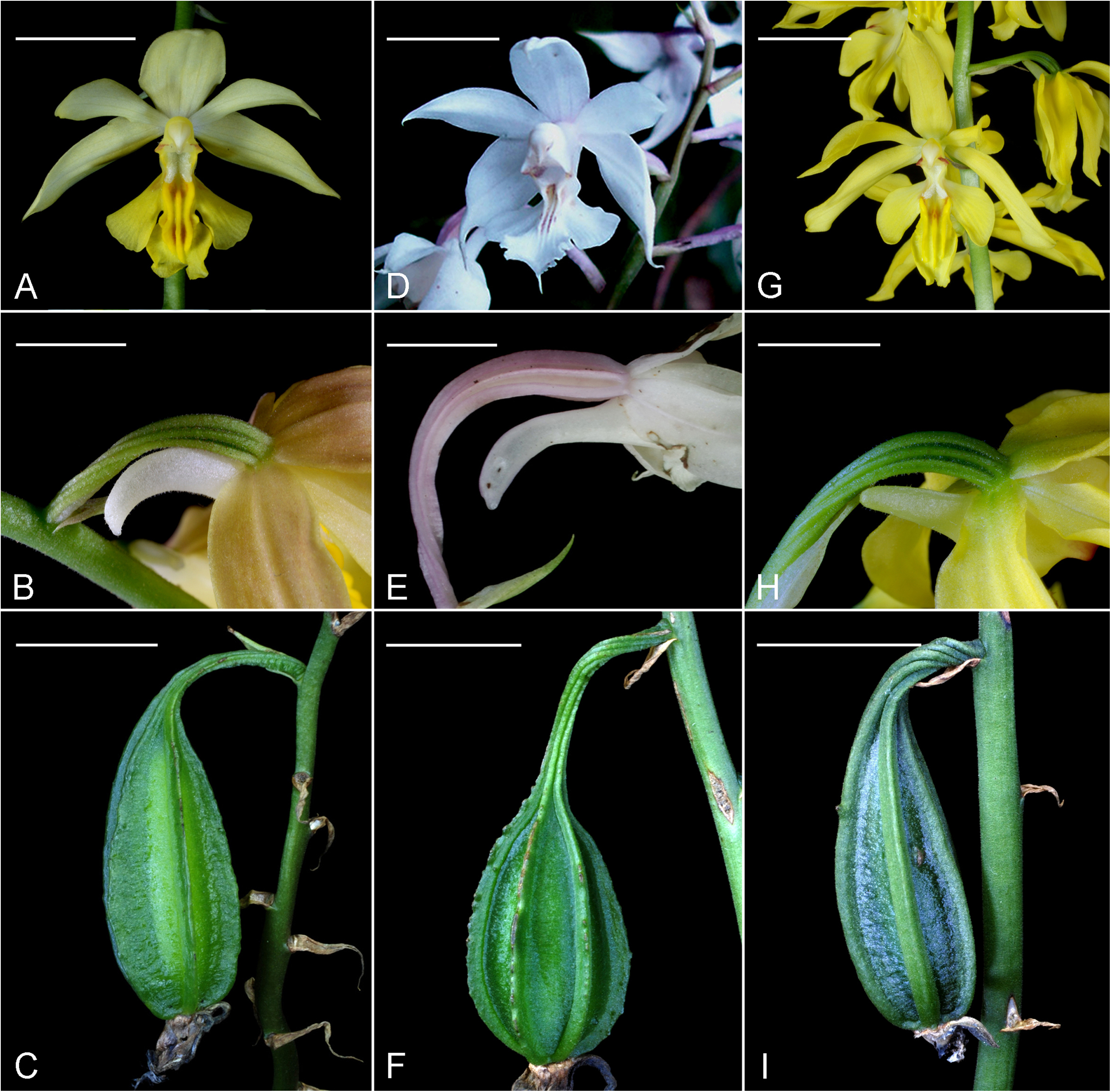

Supplement: Supplementary file 2 — Authors’ original file for figure 2 [file 40529_2011_22_MOESM2_ESM.tif]

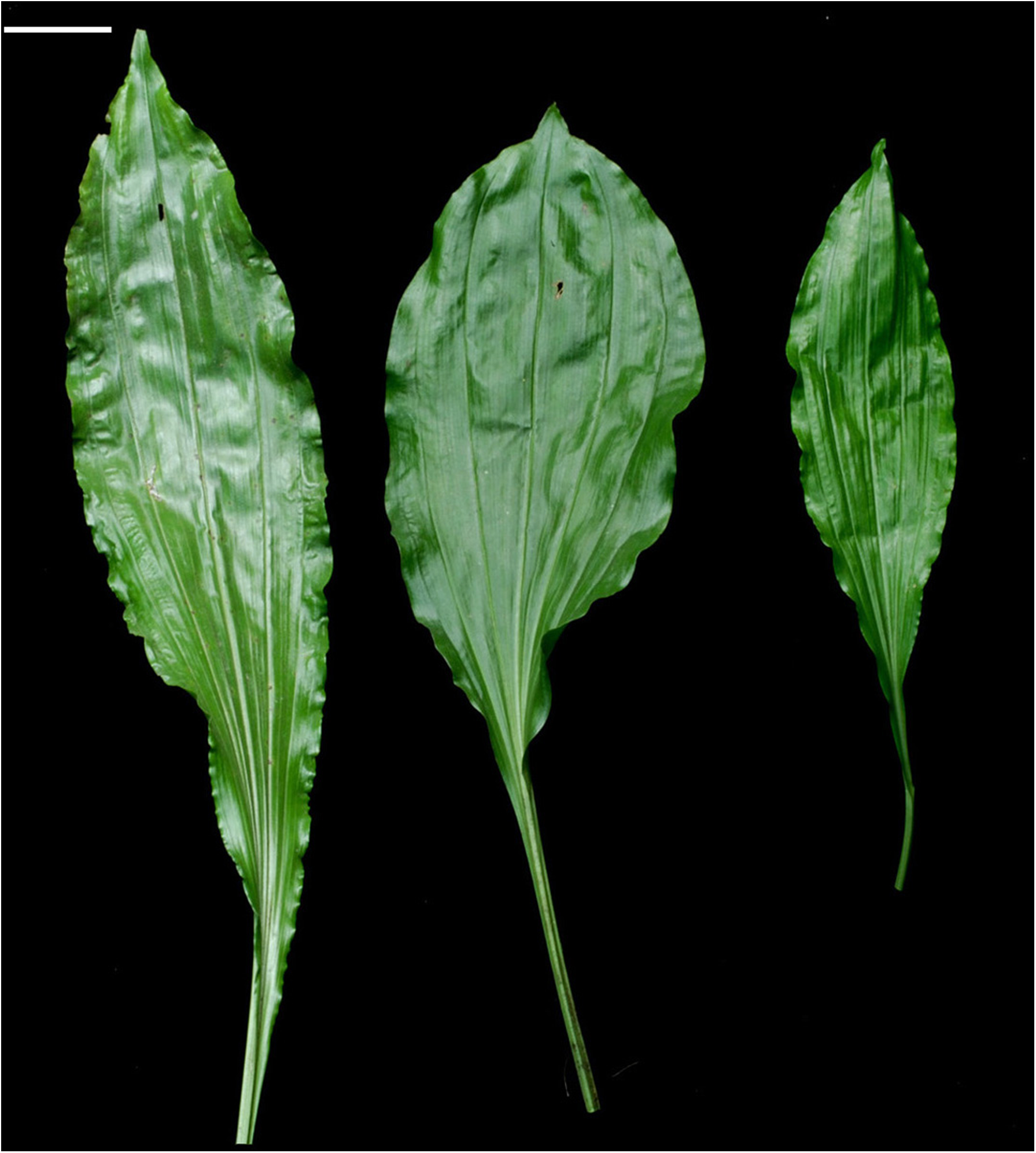

Supplement: Supplementary file 3 — Authors’ original file for figure 3 [file 40529_2011_22_MOESM3_ESM.tif]

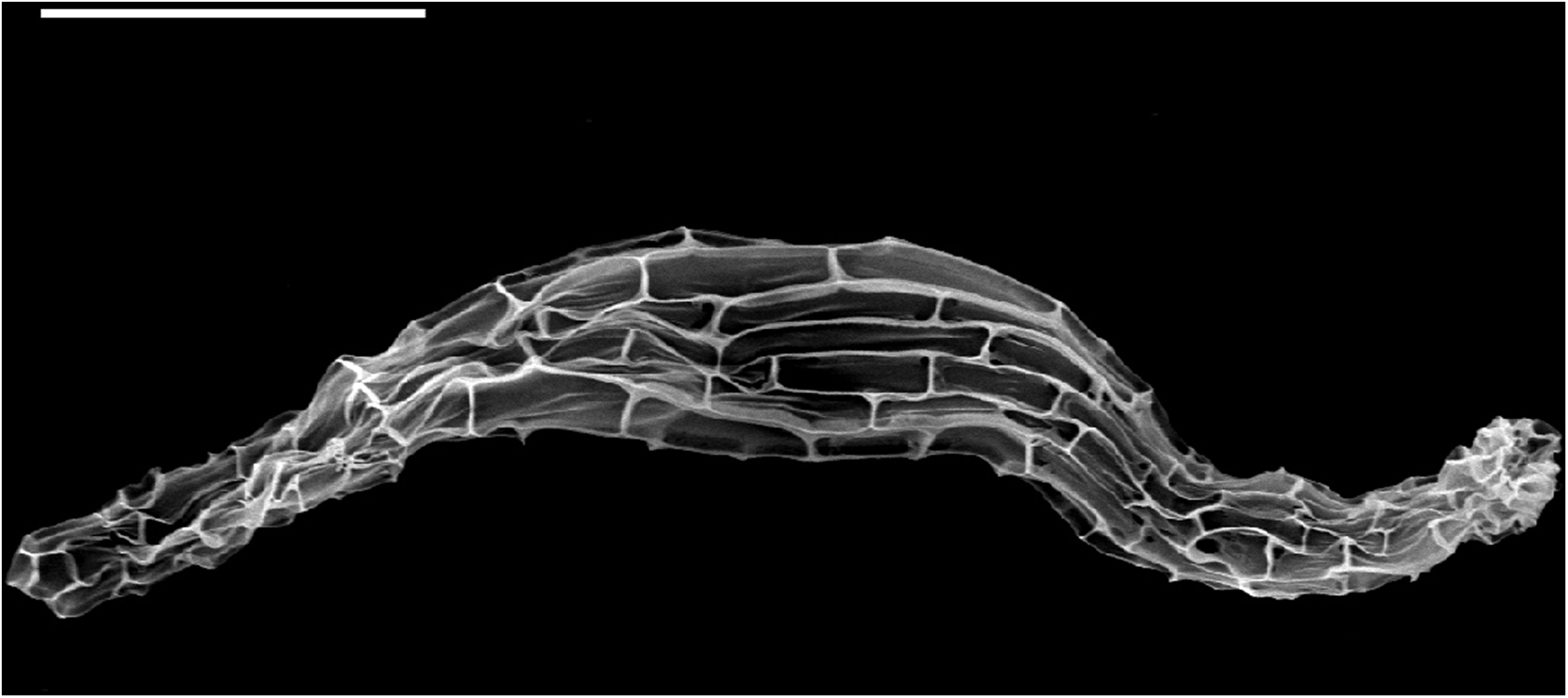

Supplement: Supplementary file 4 — Authors’ original file for figure 4 [file 40529_2011_22_MOESM4_ESM.tif]

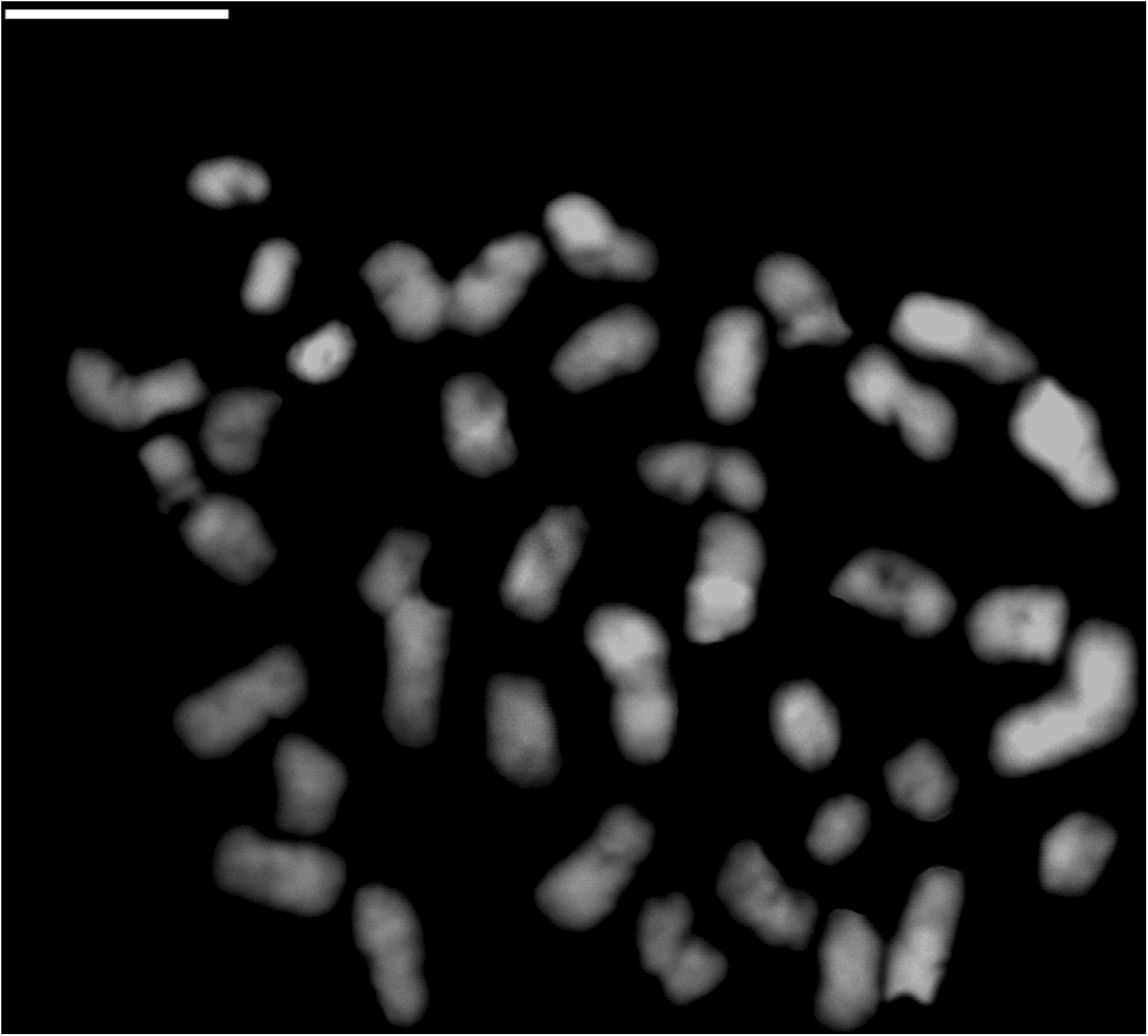

Supplement: Supplementary file 5 — Authors’ original file for figure 5 [file 40529_2011_22_MOESM5_ESM.tif]

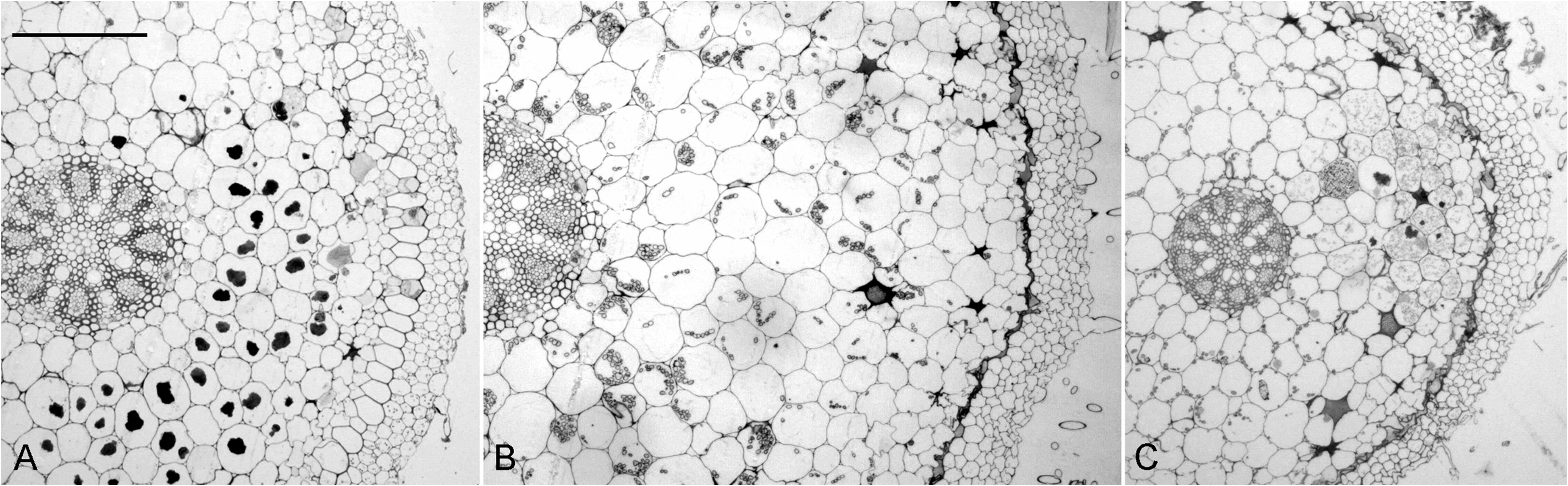

Supplement: Supplementary file 6 — Authors’ original file for figure 6 [file 40529_2011_22_MOESM6_ESM.tif]
